# ITS as an environmental DNA barcode for fungi: an *in silico *approach reveals potential PCR biases

**DOI:** 10.1186/1471-2180-10-189

**Published:** 2010-07-09

**Authors:** Eva Bellemain, Tor Carlsen, Christian Brochmann, Eric Coissac, Pierre Taberlet, Håvard Kauserud

**Affiliations:** 1 National Centre for Biosystematics, Natural History Museum, University of Oslo, P.O. Box 1172 Blindern, NO-0318 Oslo, Norway; 2 Microbial Evolution Research Group (MERG), Department of Biology, University of Oslo, P.O. Box 1066 Blindern, N-0316 Oslo, Norway; 3 Laboratoire d'Ecologie Alpine (LECA), CNRS UMR 5553, University Joseph Fourier, BP 53, 38041 Grenoble Cedex 9, France

## Abstract

**Background:**

During the last 15 years the internal transcribed spacer (ITS) of nuclear DNA has been used as a target for analyzing fungal diversity in environmental samples, and has recently been selected as the standard marker for fungal DNA barcoding. In this study we explored the potential amplification biases that various commonly utilized ITS primers might introduce during amplification of different parts of the ITS region in samples containing mixed templates ('environmental barcoding'). We performed *in silico *PCR analyses with commonly used primer combinations using various ITS datasets obtained from public databases as templates.

**Results:**

Some of the ITS primers, such as ITS1-F, were hampered with a high proportion of mismatches relative to the target sequences, and most of them appeared to introduce taxonomic biases during PCR. Some primers, e.g. ITS1-F, ITS1 and ITS5, were biased towards amplification of basidiomycetes, whereas others, e.g. ITS2, ITS3 and ITS4, were biased towards ascomycetes. The assumed basidiomycete-specific primer ITS4-B only amplified a minor proportion of basidiomycete ITS sequences, even under relaxed PCR conditions. Due to systematic length differences in the ITS2 region as well as the entire ITS, we found that ascomycetes will more easily amplify than basidiomycetes using these regions as targets. This bias can be avoided by using primers amplifying ITS1 only, but this would imply preferential amplification of 'non-dikarya' fungi.

**Conclusions:**

We conclude that ITS primers have to be selected carefully, especially when used for high-throughput sequencing of environmental samples. We suggest that different primer combinations or different parts of the ITS region should be analyzed in parallel, or that alternative ITS primers should be searched for.

## Background

Molecular identification through DNA barcoding of fungi has, during the last 15-20 years, become an integrated and essential part of fungal ecology research and has provided new insights into the diversity and ecology of many different groups of fungi (reviewed by [1-4]). Molecular identification has made it possible to study the ecology of fungi in their dominant but inconspicuous mycelial stage and not only by means of fruiting bodies. Interest in sequenced-based analysis of environmental samples ('environmental barcoding') has increased in the past decade as it allows to study abundance and species richness of fungi at a high rate and more reliably than conventional biotic surveys (e.g. [5-10]). The internal transcribed spacer (ITS) of nuclear DNA (nrDNA) is the preferred DNA barcoding marker both for the identification of single taxa and mixed environmental templates ('environmental DNA barcoding'). It has recently been proposed as the official primary barcoding marker for fungi (Deliberation of 37 mycologists from 12 countries at the Smithsonian's Conservation and Research Centre, Front Royal, Virginia, May 2007). More than 100 000 fungal ITS sequences generated by conventional Sanger sequencing are deposited in the International Nucleotide Sequence Databases and/or other databases [11], providing a large reference material for identification of fungal taxa. However, these data are to some extent hampered by misidentifications or technical errors such as mixing of DNA templates or sequencing errors [12]. Furthermore, a large amount of partial ITS sequences generated by next-generation sequencing has recently been deposited in public sequence databases.

**Figure 1:**
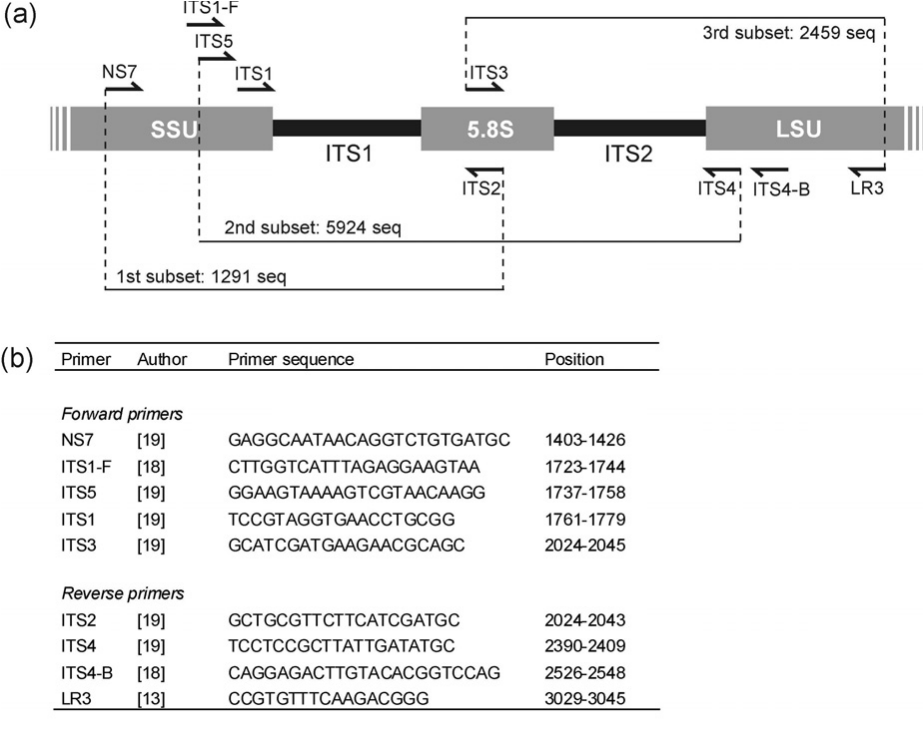
**Commonly used primers for amplifying parts or the entirety of the ITS region**. a) Relative position of the primers, design of the subsets and number of sequences in each subset. b) Primer sequences, references and position of the primer sequence according to a reference sequence of *Serpula himantioides *(AM946630) stretching the entire nrDNA repeat.

The ITS region includes the ITS1 and ITS2 regions, separated by the 5.8S gene, and is situated between the 18S (SSU) and 28S (LSU) genes in the nrDNA repeat unit (Figure 1). The large number of ITS copies per cell (up to 250; [13]) makes the region an appealing target for sequencing environmental substrates where the quantity of DNA present is low. The entire ITS region has commonly been targeted with traditional Sanger sequencing approaches and typically ranges between 450 and 700 bp. Either the ITS1 or the ITS2 region have been targeted in recent high-throughput sequencing studies [14-17], because the entire ITS region is still too long for 454 sequencing or other high-throughput sequencing methods. Using high-throughput sequencing, thousands of sequences can be analysed from a single environmental sample, enabling in-depth analysis of the fungal diversity. Various primers are used for amplifying the entire or parts of the ITS region (Figure 1). The most commonly used primers were published early in the 1990's (e.g. [18,19] when only a small fraction of the molecular variation in the nrDNA repeat across the fungal kingdom was known. Several other ITS primers have been published more recently [20] but have not been used extensively compared to the earlier published primers. However, little is actually known about the potential biases that commonly used ITS primers introduce during PCR amplification. Especially during high-throughput sequencing, where quantification (or semi-quantification) of species abundances is also possible to a certain degree (although hampered by factors like copy-number variation), primer mismatches might potentially introduce large biases in the results because some taxonomic groups are favoured during PCR. Our main focus in this study is on the two dominating taxonomic groups of fungi in the Dikarya, Ascomycota and Basidiomycota. Ascomycota represents the largest phylum of Fungi, with over 64,000 species, while Basidiomycota contains about 30,000 described species [21]. In total those two groups represent 79% of the described species of true Fungi.

The aim of this study was to analyse the biases commonly used ITS primers might introduce during PCR amplification. First, we addressed to what degree the various primers mismatch with the target sequence and whether the mismatches are more widespread in some taxonomic groups. Second, we considered the length variation in the amplified products, in relation to taxonomic group, to assess amplification biases during real (*in vitro*) PCR amplification, as shorter DNA fragments are preferentially amplified from environmental samples containing DNA from a mixture of different species [22]. Finally, we analyzed to what degree the various primers co-amplify plants, which often co-occur in environmental samples. For these purposes we performed *in silico *PCR using various primer combinations on target sequences retrieved from EMBL databases as well as subset databases using the bioinformatic tool EcoPCR [23]. In order to better simulate real PCR conditions, we allowed a maximum of 0 to 3 mismatches except for the 2 last bases of each primer and we assessed the melting temperature (Tm) for each primer in relation to primer mismatches.

## Methods

### Compilation of datasets

The EcoPCR package contains a set of bioinformatics tools developed at the Laboratoire d'Ecologie Alpine, Grenoble, France ([23], freely available at http://www.grenoble.prabi.fr/trac/ecoPCR). The package is composed of four pieces of software, namely 'ecoPCRFormat', 'ecoFind', 'ecoPCR' and 'ecoGrep'. Briefly, EcoPCR is based on the pattern matching algorithm agrep [24] and selects sequences from a database that match (exhibit similarity to) two PCR primers. The user can specify (1) which database the given primers should be tested against, and (2) the primer sequences. Different options allow specification of the minimum and maximum amplification length, the maximum count of mismatched positions between each primer and the target sequence (excluding the two bases on the 3'end of each primer), and restriction of the search to given taxonomic groups. The ecoPCR output contains, for each target sequence, amplification length, melting temperature (Tm), taxonomic information as well as the number of mismatched positions for each strand.

First, we retrieved from EMBL sequences from fungi in the following categories: 'standard', 'Genome sequence scan', 'High Throughput Genome sequencing', 'Whole Genome Sequence' from ftp://ftp.ebi.ac.uk/pub/databases/embl/release/ (release embl_102, January 2010) to create our initial database. It corresponds to 1,212,954 sequences including approximately 79,500 ITS sequences (estimated from EMBL SRS website requesting for fungi sequences annotated with 'ITS' or 'Internal Transcribed Spacer'). These ITS entries refer to more than 10,800 taxa. This database hereafter referred to as the "fungi database" was compiled using EcoPCRFormat.

To assess the specificity of the primers to fungi, we used the plant database from EMBL (release embl_102, January 2010 from ftp://ftp.ebi.ac.uk/pub/databases/embl/release/) to run amplifications using the same primers as for fungi. This database, hereafter referred to as the "plant database", contained 1,253,565 sequences, including approximately 65,000 ITS sequences (estimated from EMBL SRS website requesting for viridiplantae sequences annotated with 'ITS' or 'Internal Transcribed Spacer'). These ITS entries refer to more than 6,100 taxa. This database was also compiled using EcoPCRFormat.

As there are relatively few sequences submitted to public databases covering the entire ITS region as well as the commonly used universal primer sites in the flanking SSU and LSU regions, we created three subset datasets covering either ITS1, ITS2 or the entire ITS region. From the initial fungi database, we compiled three subset databases (hereafter referred to as subset 1, 2, and 3) by *in silico *amplification (see below) of target sequences using the following primer pairs: NS7-ITS2 (dataset 1, focused on ITS1 region), ITS5-ITS4 (dataset 2, including both ITS1 and ITS2 regions) and ITS3-LR3 (dataset 3, focused on ITS2 region). To simulate relatively stringent PCR conditions, a single mismatch between each primer and the template was allowed except in the 2 bases of the 3' primer end. These three subsets were then compiled using EcoPCRFormat and included 1291, 5924 and 2459 partial nrDNA sequences, respectively.

### *In silico *amplification and primer specificity to fungi

Using EcoPCR, we ran *in silico *amplifications from both the fungi and the plant databases using various commonly used primer combinations, to assess the number of amplifications and the specificity of the primers to fungi. For each amplification, we allowed from 0 to 3 mismatches between each primer and the template (excluding mismatches in the 2 bases of the 3' primer end) in order to simulate different stringency conditions of PCRs. Secondly, from the three subsets, we amplified sequences using different internal primer combinations in order to evaluate the various primers (Figure 1). From dataset 1 we used the primer combinations ITS1-F-ITS2, ITS5-ITS2 and ITS1-ITS2. From dataset 2 we used the combinations ITS1-ITS4 (amplifying both ITS1 and ITS2 introns), ITS3-ITS4 and ITS5-ITS2. From dataset 3 we used the combinations ITS3-ITS4 and ITS3-ITS4B. During these virtual PCRs we also allowed from 0 to 3 mismatches between each primer and the template, except in the 2 bases of the 3' primer end.

### Assessing the degree of primer mismatches and Tm

For all *in silico *internal amplifications from each subset, we assessed the proportion of sequences retrieved when allowing for 0 to 3 mismatches between each primer and the template. For the amplifications from each subset, we used an external primer (one of the primers used to create the subset) and an internal primer. Therefore, for each analysis, we assessed the proportion of sequences including mismatches for the internal primer only. The primer pair ITS5-ITS2 was evaluated both for subset 1 and subset 2, with the focus on ITS5 for subset 1 and on ITS2 for subset 2 (as those primers correspond to internal primers within their respective subsets). Similarly, the primer pair ITS3-ITS4 was evaluated both for subsets 2 and 3, with the focus on ITS3 in subset 2 and ITS4 in subset 3. The primer ITS1 was evaluated both for subset 1 (with the combination ITS1-ITS2) and for subset 2 (with the combination ITS1-ITS4) as ITS2 and ITS4 were used as external primers in subsets 1 and 2, respectively.

To assess whether certain taxonomic groups were more prone to mismatches, we assessed the proportion of sequences including one mismatch for each of the three taxonomic groups 'ascomycetes', 'basidiomycetes' and 'non-dikarya' (the latter is a highly polyphyletic group including e.g. Blastocladiomycota, Chytridiomycota, Glomeromycota and Zygomycota [25]). We also assessed the Tm for each primer based on the analyses from internal amplifications, allowing a single mismatch. The Tm is defined as the temperature at which half of the DNA strands are in the double-helical state and half are in the "random-coil" states. The strength of hybridization between the primers and the template affects Tm. It is therefore informative to assess how Tm decreases as the number of mismatches increases, i.e. with less stringent PCR conditions. Tm was calculated in ecoPCR based on a thermodynamic nearest neighbor model [26]. Exact computation was performed following [27].

### Assessing bias in amplification length relative to taxonomic group

To further assess the taxonomic bias introduced by the use of the different primer pairs, we separated the amplified sequences from selected analyses into the groups 'ascomycetes', 'basidomycetes' and 'non-dikarya' based on their taxonomic identification number, using the ecoGrep tool. These selected analyses were (1) the three subsets, and (2) all internal amplifications within each subset with one mismatch allowed. The amplification length was reported for each analysis.

## Results

### Relative amplification of different primer combinations from the fungi and plant databases

The number of fungal versus plant sequences amplified *in silico *with various ITS primer combinations directly from the raw data downloaded from EMBL (Table 1) mainly reflected the number of sequences deposited. However, the number of amplified sequences varied considerably with varying stringency conditions (in this context allowing zero to three mismatches) across different primer combinations (see Table 1 for details). Only a few plant ITS sequences were amplified using the fungus-specific primer ITS1-F (ranging from 20 to 24 sequences under different stringency conditions). Assessing these sequences using Blast, 20 out of 24 were revealed to be fungal sequences erroneously deposited as algae from an unpublished study (six *Liagora *species, two *Caulerpa *species, *Helminthocladia australis*, and *Ganonema farinosum*). There was a sequence deposited as *Chorella *matching a fungal sequence. The three others were *Chlorarachniophyte *species that did not match any known fungal sequence. Some of the other primer combinations, including ITS1-ITS2, amplified a high number of plant sequences from different orders. We also confirmed that the assumed basidiomycete-specific primer ITS4-B did not amplify any plant sequences even when allowing 3 mismatches.

**Table 1:** Number of plant and fungi ITS sequences amplified *in silico *from EMBL fungal and plant databases, using the various primer combinations and allowing none to three mismatches.

Primer comb.	Fungal ITS sequences				Plant ITS sequences			
Number of mismatches *	**0**	**1**	**2**	**3**	**0**	**1**	**2**	**3**
	
ITS5-ITS4	5482	5924	6026	6123	500	514	5667	5996
NS7-ITS2	1067	1291	1313	1320	23	190	231	403
ITS3-LR3	2070	2459	2499	2548	51	168	248	300
ITS1-ITS2	17545	19816	25223	25457	1107	17665	18755	19084
ITS1-F-ITS2	2112	4169	4592	4658	20	21	21	24
ITS5-ITS2	7713	8993	9180	9293	94	703	11123	12100
ITS1-ITS4	10013	10610	12488	12656	5783	6740	7500	7620
ITS3-ITS4	18815	21195	21663	22078	415	7829	8583	8852
ITS3-ITS4-B	1269	1673	1811	1863	0	0	0	0

* The number of mismatches allowed between the primer and the DNA strand reflects the stringency level of the PCR, i.e. strict PCR conditions such as annealing temperature close to or above the recommended Tm will not allow unspecific sequences (including one or more mismatches) to be amplified.

### Primer mismatches in sequence subsets

**Figure 2:**
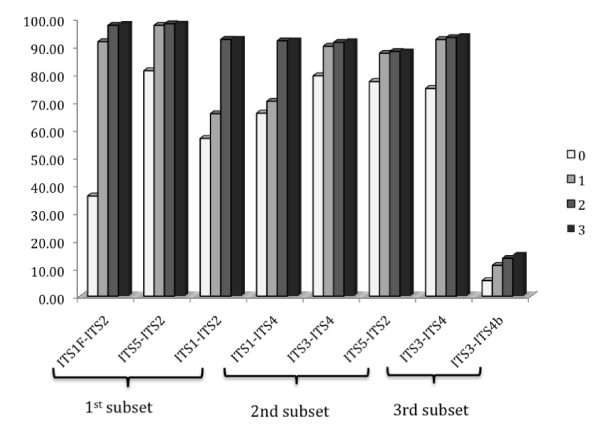
**Percentage of sequences amplified from each subset using different primer pairs allowing a maximum of 0, 1, 2, or 3 mismatches**.

The selected ITS primers showed large variation in their ability to amplify fungal sequences from the three subsets when allowing different number of mismatches (Figure 2). All primer pairs amplified at least 90% of the sequences when allowing two or three mismatches, with the exception of ITS4-B (see below). It is noteworthy that the percentages of sequences were quite similar for two and three mismatches, indicating that rather few sequences included three mismatches. Under strict conditions (i.e. allowing no mismatches), the proportion of amplified sequences varied considerably between primer pairs, ranging from 36% for ITS1-F to 81% for ITS5 (Figure 2).

Allowing one mismatch increased the proportion of amplified sequences from 36% to 91.6% for the commonly used primer ITS1-F, implying that more than half of the amplified sequences included one mismatch. ITS5 amplified the highest proportion of the sequences when allowing for a single mismatch (97.5%), and less than 10% of the sequences in each taxonomic group included one mismatch. The primer ITS1, on the other hand, only amplified 56.8% and 65.9% of the sequences from subsets one and two, respectively, when allowing no mismatches. Allowing three mismatches, ITS1 was still only able to amplify 92% of the sequences in subsets one and two. Allowing no mismatches, the complementary primers ITS2 and ITS3 amplified 79.4% and 77.3% of all sequences respectively, in subset 2. Allowing one mismatch, these numbers increased to 87.5 and 90%, respectively. Primer ITS4 amplified 74.9% of all sequences in subset 3 and this proportion only increased to 93.7% when allowing three mismatches. The assumed basidiomycete-specific primer ITS4-B amplified only 5.6% of the sequences in subset 3 under strict conditions (corresponding to 46% of the basidiomycetes sequences, see below) and up to 14.9% allowing 3 mismatches. However, about half of the sequences included a mismatch when a single mismatch was allowed.

### Taxonomic bias for different primers

The taxonomic composition in the three target sequence subsets (Figure 1) was compared with the taxonomic composition in the amplified datasets in order to reveal whether a taxonomic bias was introduced during the amplification process (Table 2). A single mismatch was allowed during these comparisons. The primers ITS1, ITS1-F and ITS5 amplified a notably higher proportion of basidiomycetes in subset 1. In contrast, primers ITS2, ITS3 and ITS4 (the two first being complementary) were biased towards ascomycetes when analysing subsets 2 and 3. The assumed basidiomycete-specific primer combination ITS3-ITS4-B only amplified 39.3% of the basidomycete sequences. Primers ITS4 and ITS5 amplified the highest proportion of 'non-dikarya' sequences. The number of mismatches allowed had a significant impact on the optimal annealing temperature to be used in the PCR reaction (Table 3). Optimal annealing temperatures decreased by approximately 6-8 degrees Celsius with each additional mismatch.

**Table 2:** Percentage of sequences amplified *in silico*, allowing one mismatch, from ascomycetes, basidiomycetes and 'non-Dikarya' with different primer combinations and using the three sequence subsets 1-3 (see Material and Methods) as templates.

Data subsets	Primer comb.	Ascomycetes	Basidiomycetes	'non-Dikarya'
Subset 1	ITS1*-ITS2	61.21	86.21	88.57
	ITS1-F*-ITS2	90.75	99.14	92.38
	ITS5*-ITS2	90.84	99.14	98.10
Subset 2	ITS1*-ITS4	61.91	82.00	84.86
	ITS3*-ITS4	98.39	73.91	91.04
	ITS5-ITS2*	94.89	72.10	92.63
Subset 3	ITS3-ITS4*	94.71	85.55	98.49
	ITS3-ITS4-B*	-	39.31	-

* primer evaluated for mismatches within each pair

**Table 3:** Melting temperature (Tm) of each primer according to the number of mismatches allowed between the primer and the target sequence.

**Primer**	**Number of mismatches allowed**			
	**0**	1*	2*	3*
	
ITS1(1) **	58.64	51.75+/-2.88	46.51+/-0.6	41.4+/-NA
ITS1(2) **	58.64	52.02+/-2.58	46.46+/-0.87	39.49+/-2.75
ITS1-F	51.04	42.31+/-1.2	38.91+/-2.62	31.64+/-0.67
ITS2	56.68	48.5+/-1.97	39.3+/-2.74	32.99+/-5.67
ITS5	51.64	41.8+/-1.69	36.6+/-3.93	NA
ITS3	56.68	50.6+/-1.15	44.3+/-3.65	39.93+/-7.25
ITS4	50.9	45.04+/-1.3	35.94+/-3.38	32.73+/-1.83
ITS4-B	59.33	54.49+/-2.39	46.6+/-3.06	37.72+/-7.38

* Mean Tm +/- SD is given for primers with 1 or more mismatches as the Tm depends on the type of mismatch.

** ITS1 is evaluated both with the first subset (1) and the second subset (2).

### Taxonomic bias relative to length of the amplified region

**Figure 3:**
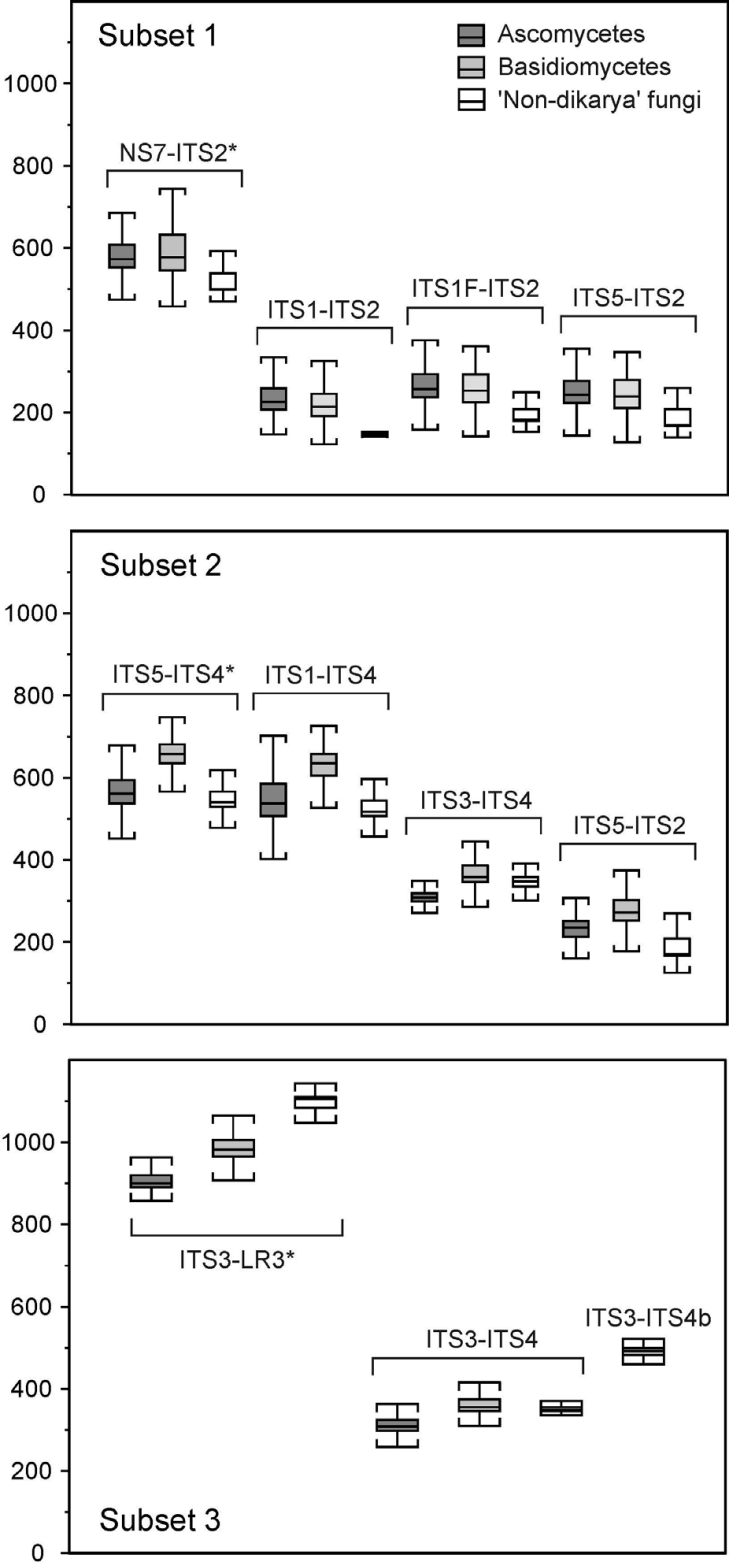
**Box plots illustrating length differences between the amplicons obtained using different primer combinations for each of the three subsets**. The plot in each subset represents the primer pair used to create the subset (*).

We found considerable length variation among the amplified fragments both in the ITS1 and ITS2 regions, as well as in the entire ITS region (Figure 3). A taxonomic bias in relation to length was apparent but not consistent between the ITS regions. In the ITS1 region, the proportions of ascomycetes and basidiomycetes were quite similar across the size range (p = 0.2, two tailed T-test), but 'non-dikarya' fungi had far more short fragments and differed significantly from the two other groups (p < 0.01 and p < 0.01, two-tailed T-tests). In contrast, in the ITS2 region, the proportion of ascomycetes and basidiomycetes were highly skewed across the size range, with basidiomycetes having significantly longer ITS2 fragments than ascomycetes (p < 0.01, two-tailed T-test; on average 95.2 bp longer fragments). Also for the entire ITS region (primer pair ITS1-ITS4), basidiomycetes had significantly longer fragments than ascomycetes (p < 0.01, two-tailed T-test), with average lengths of 634.9 versus 551.0 bp, respectively. The 'non-dikarya' fungi had significantly shorter ITS fragments than the basidiomycetes (p < 0.01, T-test), but did not differ significantly from the ascomycetes (p = 0.34, two-tailed T-test).

## Discussion

Although the ITS region has been widely used as a genetic marker during the last 15 years for exploring fungal diversity in environmental samples (e.g. [7,8,10,28]), little effort has been invested to explore the potential biases that the most commonly used ITS primers may introduce during PCR. In this study we have documented how the most commonly used fungal ITS primers are hampered by different types of biases (length bias, taxonomic bias and primer mismatch bias). Hence, in environmental sequencing studies aiming at describing fungal diversity and community composition these primers should be used with caution. Our analyses were based on entries in the public sequence databases (GenBank, EMBL and DDBJ). A general but naive assumption in studies based on this type of data is that the sequences are reliable from a technical aspect and that the sequenced samples have been correctly identified taxonomically. However, these two assumptions are often violated. Given that the quality control of the raw data typically depends solely on the scientist depositing the sequences, a proportion of published sequences admittedly contains errors [29]. In addition, Nilsson et al. [12] showed that about 20% of the fungal DNA sequences from the public sequence databases may be identified to incorrect species, and that the majority of entries lack descriptive and up-to-date annotations. However, our analyses deal with taxonomic groups at the sub-kingdom/phylum level (basidiomycetes, ascomycetes and 'non-dikarya fungi') and it is unlikely that those classes suffer significantly from incorrect identifications (e.g. that ascomycetes have been accessioned as basidiomycetes). The fact that no ascomycete sequences were amplified using primer ITS4-B, even when allowing 3 mismatches (Table 1), also supports the reliability of the conclusions in this respect. All the investigated primers were hampered by some mismatches relative to the target sequences in subsets 1-3, and they also varied in their specificity to fungi versus plants. It is noteworthy that ITS1-F, which is frequently used in fungal environmental sequencing studies and assumed to be fungal specific [18], only amplified three plant sequences after removing the fungal sequences erroneously deposited as plants. Those three sequences deposited as plants most probably corresponded to errors as well. However, the ITS1-F primer is hampered with a high degree of mismatches. Our analysis indicates that it may be important to use this primer under relaxed PCR conditions when targeting all fungi in an environmental sample. We confirmed that the primer ITS4-B, which has also often been used in environmental sequencing studies (e.g. [8,28,30,31]), is very specific to basidiomycetes, as it did not amplify plant ITS even under relaxed PCR conditions. However, this primer is only able to target a small proportion of the basidiomycete diversity (Table 4). Mainly Boletales and a fraction of the Agaricales are amplified under strict conditions, while under relaxed conditions, Chantharellales, Hymenochaetales, Tremellomycetes, Polyporales and Russulales are amplified to a certain degree (from 28 to 94% depending on the group). Pucciniomycotina and Ustilaginomycotina are not amplified at all. Hence, our *in silico *analyses indicate that ITS4-B should be used with great caution or perhaps abandoned completely in environmental sequencing studies where the aim is to characterize the diversity of all basidiomycetes. Although not specific to fungi, the primer pairs ITS5-ITS2 and ITS3-ITS4 apparently have a better ability to amplify fungal ITS as the proportion of sequences amplified does not vary much between strict and relaxed PCR conditions. Overall, the results indicate that it is important to assess the specificity of the amplification in relation to PCR stringency before interpreting the results from environmental samples in terms of abundance and diversity.

**Table 4:** Number (percentages) of sequences and amplified in each of the most common Basidiomycete groups, from the original subset3 and from the amplification of ITS3-ITS4-B from subset3, allowing no or 3 mismatches.

	subset3	ITS3-4B_3mis	ITS3-4B_0mis
Agaricales	361	269 (74.5)	118 (32.7)
Boletales	18	17 (94.4)	15 (83.3)
Cantharellales	33	31 (93.9)	0
Hymenochaetales	10	7 (70)	0
Polyporales	28	8 (28.6)	0
Russulales	97	64 (66.0)	0
Thelephorales	6	4 (66.7)	0
Dacrymycetes	1	0	0
Tremellomycetes	38	13 (34.2)	0
Pucciniomycotina	8	0	0
Ustilaginomycotina	21	0	0
Other categories *	71	21 (29.6)	3 (4.2)

* 'Other categories' represent smaller orders including Agaricomycetidae.

Our *in silico *analyses further indicate that most of the primers will introduce a taxonomic bias due to higher levels of mismatches in certain taxonomic groups. When allowing one mismatch (corresponding to rather stringent PCR conditions) we found that the primer pairs ITS1-F, ITS1 and ITS5 preferentially amplified basidiomycetes whereas the primer pairs ITS2, ITS3 and ITS4 preferentially amplified ascomycetes. This type of bias must also be considered before selecting primer pairs for a given study. Also in molecular surveys of protistan and prokaryotic diversity, it has been documented that different 16S primers target different parts of the diversity [32-34].

In addition, our results clearly demonstrate that basidiomycetes, on average, have significantly longer amplicon sequences than ascomycetes both for the whole ITS region, and the ITS2 region. This fact probably also introduces taxonomic bias during PCR amplification of environmental samples, since shorter fragments are more readily amplified compared to longer ones. In several studies, it has been demonstrated that a greater proportion of the diversity can be detected with short target sequences compared to longer ones [35,36]. Hence, using the ITS2 region or the whole ITS region, a higher number of the ascomycetes will probably be targeted compared to basidiomycetes. This bias could be avoided by using primers amplifying ITS1 only, but this would imply a preferential amplification of the 'non-dikarya' fungi.

## Conclusion

The *in silico *method used here allowed for the assessment of different parameters for commonly used ITS primers, including the length amplicons generated, taxonomic biases, and the consequences of primer mismatches. The results provide novel insights into the relative performance of commonly used ITS primer pairs. Our analyses suggest that studies using these ITS primers to retrieve the entire fungal diversity from environmental samples including mixed templates should use lower annealing temperatures than the recommended Tm to allow for primer mismatches. A high Tm has been used in most studies, which likely biases the inferred taxonomic composition and diversity. However, one has to find a balance between allowing some mismatches and avoiding non-specific binding in other genomic regions, which can also be a problem.

Considering the different types of biases (specificity to fungi; mismatches; length; taxonomy), we suggest that different primer combinations targeting different parts of the ITS region should be analyzed in parallel. When dealing with single culture isolates compared to environmental samples, the choice of a primer pair to amplify ITS is less problematic because there is no 'competition' between DNA fragments of different taxonomic groups/lengths, and the DNA quality is generally higher.

This study also illustrates potential benefits of using a bioinformatics approach before selecting primer pairs for a given study. We nevertheless emphasize that an *in silico *analysis does not necessarily reflect the performance of the primers *in vitro*, since there are many other PCR parameters such as ITS copy number, amplification program, and salt and primer concentration in the PCR mix that cannot easily be simulated. This study should therefore be followed up by *in vitro *PCR analyses of the fungal ITS primers where biases are measured based on sequence output, although it will be a huge task to control and check for all types of biases that might be involved. We are currently performing further bioinformatics analyses using the tool 'ecoPrimer' (http://www.grenoble.prabi.fr/trac/ecoPrimers; Riaz et al. unpublished) to identify the most appropriate barcoding primers within the ITS region and other regions, with the intent of determining whether new ITS primers, such as those recently published by Martin and Rygiewicz [20], should replace the currently used ones.

## Authors' contributions

EB, TC and HK conceived of the study, participated in its design and coordination. EB carried out the bioinformatics analysis and drafted the manuscript. EC designed the bioinformatic tool used in this study (ecoPCR). All authors helped to draft the manuscript and approved the final manuscript.
